# Mitochondria-targeted antioxidant SkQ1 inhibits leukotriene synthesis in human neutrophils

**DOI:** 10.3389/fphar.2022.1023517

**Published:** 2022-11-24

**Authors:** Galina F. Sud’ina, Ekaterina A. Golenkina, Anastasia S. Prikhodko, Natalia D. Kondratenko, Tatjana V. Gaponova, Boris V. Chernyak

**Affiliations:** ^1^ Belozersky Institute of Physico-Chemical Biology, Lomonosov Moscow State University, Moscow, Russia; ^2^ Faculty of Bioengineering and Bioinformatics, Lomonosov Moscow State University, Moscow, Russia; ^3^ National Research Center for Hematology, Russia Federation Ministry of Public Health, Moscow, Russia

**Keywords:** neutrophil, mitochondria, intracellular calcium, 5-lipoxygenase, leukotriene B4

## Abstract

Leukotrienes are among the most potent mediators of inflammation, and inhibition of their biosynthesis, is becoming increasingly important in the treatment of many pathologies. In this work, we demonstrated that preincubation of human neutrophils with the mitochondria targeted antioxidant SkQ1 (100 nM) strongly inhibits leukotriene synthesis induced by three different stimuli: the Ca^2+^ ionophore A23187, the chemotactic formyl-peptide fMLP in combination with cytocholasin B, and opsonized zymosan. The SkQ1 analogue lacking the antioxidant quinone moiety (C12TPP) was ineffective, suggesting that mitochondrial production of reactive oxygen species (ROS) is critical for activating of leukotriene synthesis in human neutrophils. The uncoupler of oxidative phosphorylation FCCP also inhibits leukotriene synthesis, indicating that a high membrane potential is a prerequisite for stimulating leukotriene synthesis in neutrophils. Our data show that activation of mitogen-activated protein kinases p38 and ERK1/2, which is important for leukotriene synthesis in neutrophils is a target for SkQ1: 1) the selective p38 inhibitor SB203580 inhibited fMLP-induced leukotriene synthesis, while the ERK1/2 activation inhibitor U0126 suppressed leukotriene synthesis induced by any of the three stimuli; 2) SkQ1 effectively prevents p38 and ERK1/2 activation (accumulation of phosphorylated forms) induced by all three stimuli. This is the first study pointing to the involvement of mitochondrial reactive oxygen species in the activation of leukotriene synthesis in human neutrophils. The use of mitochondria-targeted antioxidants can be considered as a promising strategy for inhibiting leukotriene synthesis and treating various inflammatory pathologies.

## Introduction

Neutrophils are the first to respond to infections and tissue damage. Neutrophils generate and release leukotrienes, first of all, leukotriene B4 (LTB4), which makes neutrophil’s contribution to the inflammatory state (Peters-Golden and Henderson 2007). Leukotriene B4-mediated recruitment of leukocytes and production of inflammatory cytokines are critical in chronic inflammation and sepsis. Serum LTB4 concentrations are elevated in sepsis and contribute to endothelial disorders. LTB4-mediated swarming and clustering results in severe capillaritis and lethal fungal sepsis; but tissue injury is minimized by blockade of the leukotriene LTB4 (Lee et al., 2018). In our body neutrophils are the most reach source of physiologically active compounds, leukotrienes. 5-LOX is mostly expressed in neutrophils and monocyte/macrophages. These cells produce LTB4 to stimulate chemotaxis of phagocytes to inflammatory loci. LTB4 is an important mediator of neutrophilic inflammation (Kim et al., 2006; Okunishi and Peters-Golden 2011; Tan and Weninger 2017). LTB4 from neutrophils can lead to increased local LTB4 production, neutrophil recruitment and swarming, and act as a cell-to-cell signaling molecule (Nemeth and Mocsai 2016; Subramanian et al., 2017).

LTB4 is formed from arachidonic acid under the action of the enzymes 5-lipoxygenase (5-LOX) and leukotriene A4 hydrolase (Samuelsson and Funk 1989). 5-LOX is the key enzyme in the activation of leukotriene synthesis, and many factors work in concert to stimulate 5-LOX activity. The enzyme 5-LOX binds calcium (Hammarberg and Radmark 1999; Hammarberg et al., 2000) and leukotriene synthesis is potently stimulated by an increase of intracellular calcium [(Ca^2+^)_i_]. Calcium also protects 5-LOX from inhibiting action of glutathione peroxidases (GPx) (Burkert et al., 2003). Phosphorylation of 5-LOX at Ser 271 and Ser 663 by p38 kinase dependent MAPKAP kinases (Werz et al., 2000a) and ERK1/2 (Werz et al., 2002) is important for the enzyme activation, inducing translocation of 5-LOX to the nuclear membrane and formation of multi-enzyme complex responsible for the synthesis of leukotrienes (Mandal et al., 2008; Bair et al., 2012); phosphorylation by protein kinase A leads to the loss of enzyme activity (Luo et al., 2004).

Oxidative events play an important role in 5-LOX activation. In particular, the oxidation of Fe^2+^ to Fe^3+^ at the 5-LOX active site, which is required for enzymatic activity, is mediated by lipid hydroperoxides (Rouzer and Samuelsson 1986; Rådmark 2002). Oxidative stress (oxygen-glucose deprivation (OGD)/recovery or exogenous hydrogen peroxide) has been shown to induce 5-LOX activation in 5-LOX-transfected PC12 cells (Li et al., 2009). Exogenous hydrogen peroxide or reactive oxygen species (ROS) produced by neutrophils stimulated 5-LOX in the Epstein-Barr virus transformed B-lymphocytic cell line (Werz et al., 2000b). At the same time the excess of hydrogen peroxide has been shown to inhibit 5-LOX activity in alveolar macrophages due to depletion of ATP (Sporn and Peters-Golden 1988). These findings likely explain the paradoxical stimulation of LTB4 synthesis by the antioxidant N-acetylcysteine (NAC) or catalase in alveolar macrophages and in neutrophils (Dent et al., 1997).

Mitochondria are an important source of ROS in various cell types, but only in our previous studies (Vorobjeva et al., 2017; Vorobjeva et al., 2020) mitochondrial ROS (mtROS) were introduced into the regulation of neutrophil functions, including activation NADPH oxidase, degranulation, and the formation of extracellular traps (NETs). In that studies, the mitochondria-targeted antioxidant SkQ1 [10-(6′-plastoquinonyl) decyltriphenylphosphonium bromide] was found to prevent the activation of neutrophils by A23187 and fMLP. SkQ1 selectively accumulates in mitochondria due to the positive charge of the decyltriphenylphosphonium residue and efficiently scavenge mtROS due to the antioxidant activity of plastoquinyl residue (Antonenko et al., 2008). In this study, we found that the mitochondria targeted antioxidant SkQ1 suppresses leukotriene synthesis in human neutrophils activated by A23187, fMLP or opsonized zymosan, suggesting that mitochondrial ROS (mtROS) production is critical for 5-LOX activation.

## Materials and methods

SkQ1 (10-(6′-plastoquinonyl)decyltriphenylphosphonium bromide) was synthesized as described earlier (Antonenko et al., 2008). SkQ1 and Dodecyltriphenylphosphonium bromide (C12TPP) were kindly provided by the Institute of Mitoengineering, Lomonosov Moscow State University. Hank’s balanced salt solution with calcium and magnesium but without Phenol Red and sodium hydrogen carbonate (HBSS), Dulbecco’s phosphate-buffered saline (PBS) with magnesium but without calcium, fibrinogen from human plasma, A23187, Diamide (Azodicarboxylic acid bis (dimethylamide)), cytochalasin B and peptide N-formyl-L-methionyl-L-leucyl-l-phenylalanine (fMLP) were purchased from Sigma (Steinheim, Germany). MitoQ (Mitoquinone mesylate, S8978) was purchased from Selleck Chemicals (Houston, TX, United States). Zymosan A from *Saccharomyces cerevisiae* was purchased from Goldbio (St Louis MO, United States). Zymosan was opsonized immediately before the experiment for 30 min in fresh serum from the same donor whose blood was used for neutrophil isolation and washed by repeated centrifugation in Dulbecco’s solution. Acetoxymethyl-ester (AM) conjugated fura-2 was from Thermo Fisher Scientific (Waltham, MA United States).

### Neutrophil preparation

Human polymorphonuclear leukocytes (PMNLs) were obtained from freshly collected blood with citrate anticoagulant. Experimental and the subject consent procedures were approved by the Bioethics Committee of the Lomonosov Moscow State University, Application # 6-h, version 3, Bioethics Commission meeting # 131-d held on 31.05.2021. Leukocyte-rich plasma was prepared from donor blood by sedimentation in the presence of dextran T-500 (Pharmacosmos, Holbæk, Denmark), and granulocytes were obtained as described (Aleksandrov et al., 2006). Cell viability was tested by exclusion of trypan blue. PMNLs (96%–97% purity, 98%–99% viability) were stored at room temperature in Dulbecco’s PBS containing 1 mg/ml glucose.

### Determination of 5-LOX product synthesis in intact cells and in cell homogenate

PMNLs (1 ml × 10^7^/6 ml HBSS/HEPES) were kept 10 min at 37°C in CO_2_ incubator, then reagents were added for 30 min, as indicated, followed by adding of 5-LOX stimuli: A23187 (1 µM), or cytochalasin B (5 µM) followed by fMLP (0.1 µM), or opsonized zymosan (0.04 mg/ml). The incubation was stopped by adding of an equal volume of methanol (−18°C) with 90 ng PGB2 as internal standard. Major metabolites of 5-LOX, 5S,12R-dihydroxy-6,14-*cis*-8,10-*trans*-eicosatetraenoic acid (LTB_4_), iso-LTB_4_ (5S,12SR-all-*trans*-diHETE) (t-LTB_4_), ω-OH-LTB_4_, ω-COOH-LTB_4_ and 5S-hydroxy-6-*trans*-8,11,14-*cis*-eicosatetraenoic acid (5-HETE) were identified as previously described (Golenkina et al., 2021).

For the determination of 5-LOX product formation in cell homogenates, freshly isolated PMNLs were resuspended in Buffer A (50 mM K2HPO4, 100 mM NaCl, 2 mM EDTA, 1 mM dithiothreitol, pH 7.1) containing 0.5 mM phenylmethylsulfonyl fluoride and 60 μg/ml soybean trypsin inhibitor, as published (Coffey et al., 1992) and sonicated (3 s × 10 s) on ice. Cell sonicates were centrifuged at 400 g for 10 min at 4°C to remove unbroken cells, and immediately used. 5-LOX activity determined in the mix of Buffer B (200 mM Tris-HCl pH 7.5, 3 mM CaCl2, 1.6 mM EDTA) with 1.8 mM ATP and cell homogenate, constituting no more than 10% of the total volume, as described (Grishina et al., 2008). After 10 min at 37°C with/without SkQ1, 20 µM arachidonic acid were added to start 5-LOX. The reaction was stopped after 10 min by adding of an equal volume of methanol (−18°C) with PGB2 as internal standard, and the 5-LOX metabolites were analyzed by HPLC as described for intact cells.

### Measuring of cytosolic Ca^2+^ concentration

Cytosolic calcium concentration [(Ca^2+^)_c_] changes were detected by ratiometric assay using fluorescent fura-2 dye. Freshly isolated PMNLs (10^7^ cells/mL) were incubated with 1 µM fura-2 AM in Ca^2+^-free Dulbecco’s PBS for 30 min at 37°C. Following loading, cells were pelleted, washed once and resuspended in Dulbecco’s PBS. Immediately before the experimental procedure labeled cells were resuspend in HBSS/HEPES medium, seeded in fibrinogen-coated 96-well F-bottom plates and treated according to the experimental design at 37°C in 5% CO_2_. Changes in fluorescence emitted at 510 nm were measued when exited by both 380 nm (for Ca^2+^-free dye) and 340 nm (for Ca^2+^-bound dye) for 120 s after each stimulus injection. Manipulations were performed on a ClarioStar fluorescence microplate reader (BMG Labtech, Cary, NC, United States) and MARS data analysis software package from BMG Labtech was used to process the data obtained. Both the areas under the kinetic curves (AUC) and the amplitude of changes in calcium concentration (peak height) were analyzed.

### Western immunoblotting

PMNLs (1 × 10 ^6^ cells/mL, 0.3 ml) in HBSS/HEPES were incubated with or without 100 nM SkQ1 for 30 min at 37°C in 5% CO_2_ humidified atmosphere, then 1 µM A23187 for 10 min or 5 µM cytochalasin B (10 min) followed by 0.1 µM fMLP (10 min), or OZ (0.04 mg/ml, 10 min) were added. PMNLs were lysed in hot 2x Laemmli Sample Buffer. Equal amounts of protein (≈20 µg total protein per lane), separated onto 12% SDS polyacrylamide gels and then transferred to PVDF membranes (Amersham, United States). Membranes were probed with antibodies against p-38, phospho-p38, ERK1/2, phospho-ERK1/2 (Cell Signaling, United States, ## 9212S, 9211S, 9102S, and 9101S, respectively). Membranes were treated with goat anti-rabbit HRP-conjugated secondary antibody (Sigma, United States) and developed with ECL chemiluminescence reagents–SuperSignal™ West Dura Extended Duration Substrate (ThermoFisher, United States) according to the manufacturer’s protocol. ChemiDoc System (Bio-Rad, United States) and appropriate software were used for visualizing and blot analysis.

### Statistical analysis

To quantify leukotriene synthesis, two-way ANOVA followed by Tukey’s multiple comparisons test was performed, and graphs were plotted using GraphPad Prism version 9.2.0 software for Windows. Results are presented as mean ± SEM. Differences with *p*-value of <0.05 were considered statistically significant.

## Results

### Mitochondria-targeted antioxidants inhibit the synthesis of leukotrienes in neutrophils

**FIGURE 1:**
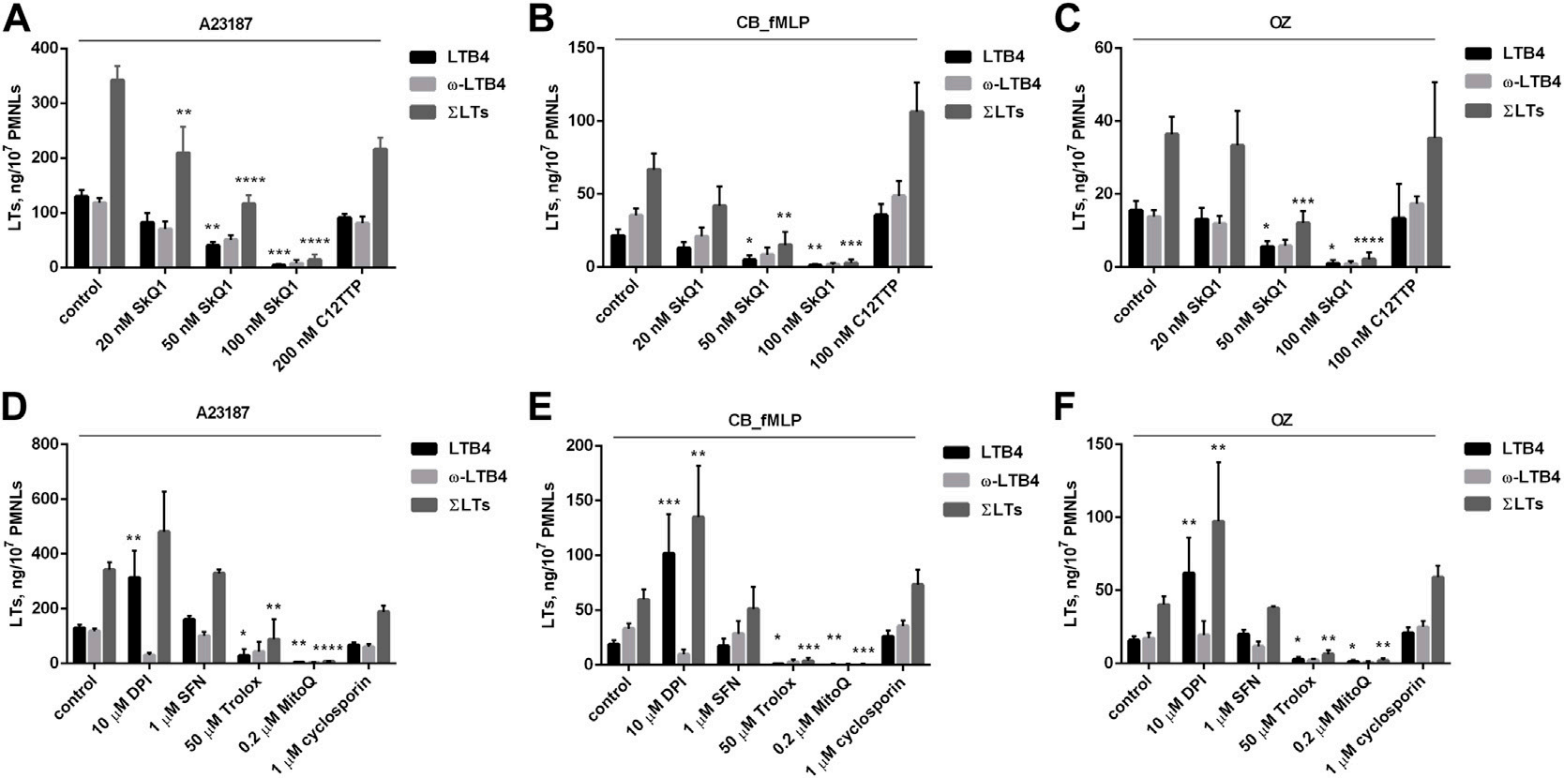
Effect of SkQ1 and C12TPP on leukotriene synthesis in human neutrophils **(A–C)**. Effect of diphenylene iodonium (DPI), sulforaphane (SFN), Trolox, MitoQ and cyclosporin A on leukotriene synthesis in human neutrophils **(D–F)**. Before treatment, PMNLs (1–1.2) × 10^7^/6 ml were pre-incubated for 10 min at 37°C at CO_2_ incubator. Then cells were treated 30 min with reagents indicated on *X*-axis. After that, A23187 (1 µM) **(A,D)**, or cytochalasin B (5 µM) **(B,E)** or OZ (0.04 mg/ml) **(C,F)** were added for 10min, then fMLP (0.1 µM) for 10 min **(B,E)**, as indicated. Values present mean ± SEM of three or more independent experiments performed in duplicate. The 5-LOX products were analyzed using HPLC, and data for LTB4, ω-OH-LTB4 and the sum of leukotrienes (ΣLTs = LTB4 + trans-LTB4 + ω-OH-LTB4) are presented. *p < 0.05, **p < 0.01, ***p < 0.001, ****p < 0.0001 for pairs of data (marked column compared with corresponding control value) by two-way ANOVA followed by Tukey’s multiple comparison test.

As shown in Figures 1A–C, the mitochondria-targeted antioxidant SkQ1 dose-dependently inhibits the synthesis of LTB4, the omega-hydroxylation product of LTB4 (ω-LTB4) and total leukotrienes (ΣLTs). This effect of SkQ1 was observed upon activation of neutrophils by three different stimuli: the Ca^2+^ ionophore A23187, the chemotactic formyl-peptide fMLP in combination with cytochalasin B (CB_fMLP), and opsonized zymosan (OZ). In all three models, 100 nM SkQ1 almost completely inhibited leukotriene synthesis, while the SkQ1 analogue lacking the antioxidant quinone moiety (C12TPP) was ineffective. SkQ1 alone, without additional stimuli, did not induce 5-LOX product synthesis in neutrophils (data not shown). Trypan blue exclusion showed no decrease in viability of PMNL cells exposed to SkQ1 (45 min, 37°C) (n = 6).

More detailed analysis of 5-LOX product synthesis, including the isomers of LTB4 (t-LTB4) and 5-HETE, induced by A23187 is shown in Supplementary Figure S1. The main metabolites formed were LTB4 and ω-LTB4, and the synthesis of t-LTB4 and 5-HETE changed in sync with them. The same pattern of products was observed upon stimulation with CB_fMLP and OZ. For not to overload the pictures, in the figures in the text we included t-LTB4 in ΣLTs and omitted the data for 5-HETE.

To test the direct effect of SkQ1 on 5-LOX in a cell-free assay, we determined the effect of SkQ1 on 5-LOX product synthesis in cell homogenates (Supplementary Figure S2). 5-HPETE/5-HETE was the predominant product in the cell-free assay. It was shown that 100 and 200 nM SkQ1, which almost completely inhibited 5-LOX product synthesis in intact cells, had no significant effect on the activity of 5-LOX in the cell homogenate. We cannot rule out that SkQ1 at higher concentrations may directly inhibit 5-LOX, but this effect is unlikely to be responsible for the action of SkQ1 in intact cells. Moreover, as we have shown earlier using the fluorescent analog of SkQ1 (Vorobjeva et al., 2017), it is almost exclusively located in mitochondria of neutrophils, while 5-LOX is activated in the cytosol and at the nuclear membrane (Radmark et al., 2007).

The data on Figures 1A–C suggest that ROS produced in mitochondria (mtROS) are required for the synthesis of leukotrienes in neutrophils. In support of this possibility, a non-targeted antioxidant, a water-soluble analogue of vitamin E (Trolox), also inhibited the synthesis of leukotrienes, although at much higher concentration than SkQ1 (Figures 1D–F). Long-chain vitamin E analogs and metabolites have previously been reported to be effective inhibitors of 5-LOX (Neukirch et al., 2021). However, short-chain analogs like Trolox were ineffective, so direct inhibition of 5-LOX by Trolox can be ruled out.

To further elucidate the role of oxidative events in the regulation of leukotriene synthesis, we used diamide, an oxidizing agent that specifically converts glutathione to its disulfide (Kosower et al., 1969). In earlier studies, diamide has been shown to stimulate 5-LOX in arachidonic acid-activated neutrophils (Hatzelmann and Ullrich 1987). Diamide only slightly stimulated the synthesis of leukotrienes in neutrophils activated by A23187, but significantly weakened the inhibitory effect of 50 nM SkQ1 (Supplementary Figure S3). Higher doses of SkQ1 almost completely inhibited leukotriene synthesis even in the presence of diamide. Higher concentrations of diamide also had an inhibitory effect in line with earlier findings (Hatzelmann and Ullrich 1987). These data are consistent with our assumption that SkQ1 inhibits leukotriene synthesis by suppressing the action of ROS.

It has been previously reported that SkQ1 stimulates the transcription factor NRF2 (nuclear factor erythroid-2-related factor 2), presumably through the activity of an electrophilic functional group in the quinone ring (Vnukov et al., 2017). NRF2 controls the expression of various antioxidant enzymes not associated with mitochondria, so it was necessary to analyze its possible role in inhibition of leukotriene synthesis by SkQ1. To do this, we applied the powerful NRF2 inducer sulforaphane (SFN) but found no effect on leukotriene synthesis (Figures 1D–F). To further explore the role of mtROS, we used one more mitochondria-targeted antioxidant MitoQ, which has less pronounced electrophilic functional groups for NRF2 activation. As shown in Figures 1D–F, this antioxidant effectively inhibits the synthesis of leukotrienes. Thus, the role of NRF2 in the effect of SkQ1 can be ruled out.

In our previous studies (Vorobjeva et al., 2017; Vorobjeva et al., 2020), we have shown that SkQ1 prevents NADPH oxidase (NOX2) activation in neutrophils activated by A23187 or fMLP. NOX2 is a powerful source of ROS in activated neutrophils; therefore, its role in the synthesis of leukotrienes was analyzed. We used an effective (albeit non-selective) NADPH oxidase inhibitor diphenylene iodonium (DPI) and did not observe inhibition of LTB4 synthesis (Figures 1D–F). These data are consistent with findings that neutrophils from patients with chronic granulomatous disease (CGD) (i.e., those with genetic defects in NOX2) produce more LTB4 than normal neutrophils, and that LTB4 causes increased neutrophilic lung inflammation in CGD mice (Song et al., 2020). As shown in Figures 1D–F, DPI significantly increased LTB4 accumulation while suppressing ω-LTB4 production under all three stimuli, indicating the inhibition of LTB4 omega-hydroxylation. Consistent with this suggestion, DPI has previously been shown to inhibit two major components that catalyze omega-hydroxylation, flavin-containing cytochrome P450 reductase and cytochrome P450 itself (Szilagyi et al., 2016).

5-LOX catalyzes the first two steps in the biosynthesis of LTB4 and other leukotrienes from arachidonic acid (AA). Production of AA from phospholipids is catalyzed by phospholipase A2 (PLA2), and both enzymes can be inhibited by SkQ1. To analyze the possible role of PLA2 in the inhibition of leukotriene synthesis by SkQ1, we measured A23187-induced leukotriene synthesis in the presence of exogenous AA. As shown in Supplementary Figure S4, the addition of AA did not affect the inhibitory effect of SkQ1. These data do not allow a conclusion about possible effect of SkQ1 on PLA2, but clearly indicate that possible changes in PLA2 activity are not responsible for the effect of SkQ1 on leukotriene synthesis. We hypothesize that activation of 5-LOX is responsible for mtROS-dependent activation of leukotriene synthesis in neutrophils.

### The effect of SkQ1 on the synthesis of leukotrienes is not mediated by changes in cytosolic Ca^2+^ concentration

**FIGURE 2:**
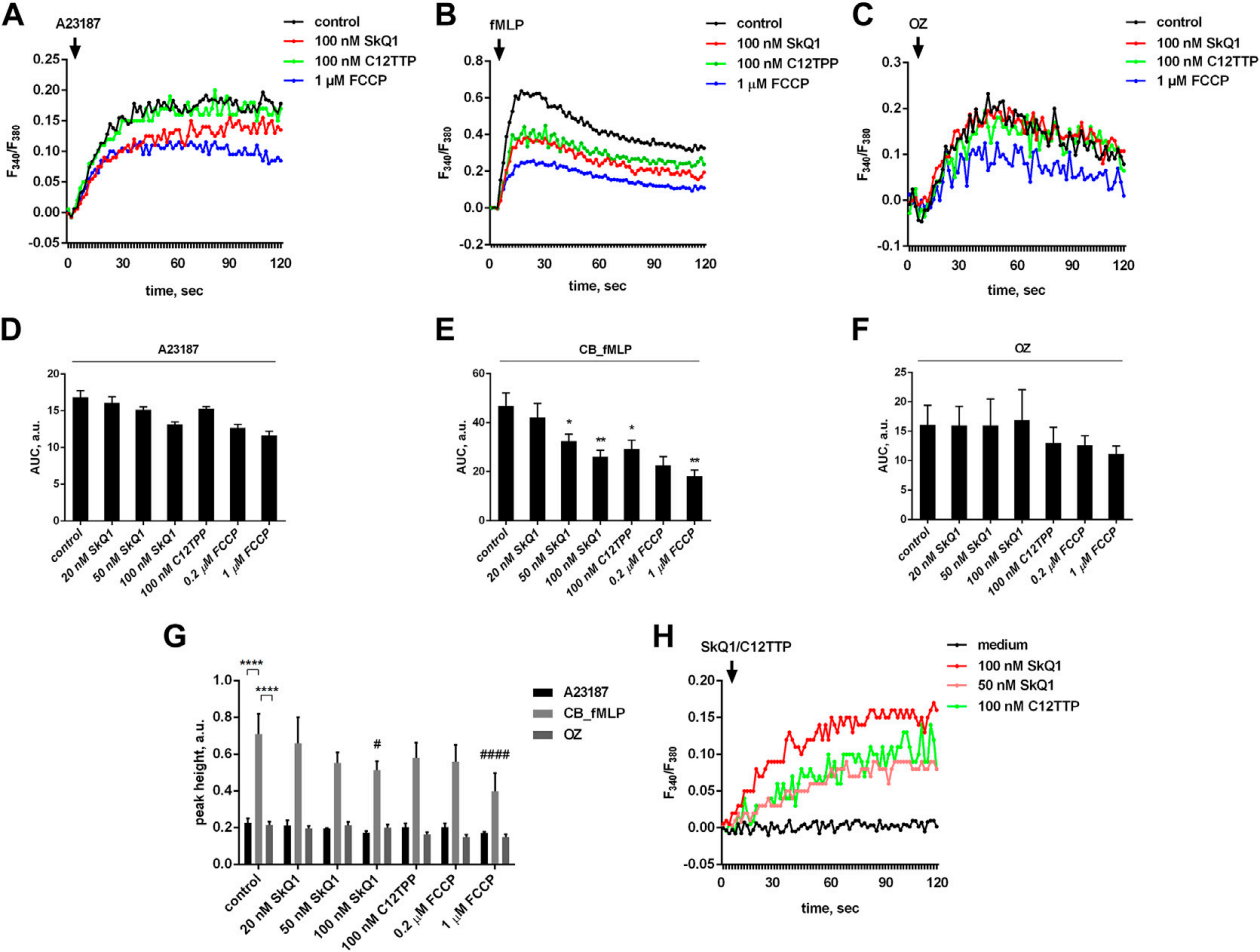
Effect of SkQ1, C12TTP and FCCP on cytosolic calcium levels in human neutrophils. PMNLs loaded with fura-2 dye (4 × 10^5^ per sample) were pre-incubated for 10 min in HBSS/HEPES medium, treated with indicated doses of SkQ1, C12TTP or FCCP for 30 min and stimulated with 1 μM A23187, sequential addition of 5 µM cytochalasin B (10 min) and 0.1 µM fMLP or OZ (0.04 mg/ml). Fluorescence intensity at 510 nm of Ca^2+^-bound and Ca^2+^-free Fura-2 was measured every second for 120 s after the addition of stimuli **(A–G)** or immediately after the addition SkQ/C12TTP (h). Changes in [Ca^2+^]_c_ are presented as typical blank corrected curves of fura-2 fluorescence ratio 335/380 **(A–C,H)**. Areas under curves within the measurement interval in arbitrary units (AUC, a. u.) are presented on **(D–F)** as means ± SEM (*n* = 4; *p < 0.05, **p < 0.01 compared to control values; by one-way ANOVA followed by Dunnett’s multiple comparisons test). Differences between the maximum and minimum values of F_340_/F_380_ (peak heights, a. u.) were assessed within the measurement interval and presented on (g) as means ± SEM (*n* = 4; ^#^ *p* < 0.05, ^####^ *p* < 0.0001, compared to corresponding control values; ****p < 0.0001 for pairs of data indicated by brackets; by two-way ANOVA followed by Tukey’s multiple comparison test).

Activation of leukotrienes synthesis depends on an increase in cytosolic Ca^2+^ concentration [(Ca^2+^)_c_] in stimulated neutrophils (Rouzer and Samuelsson 1987; Radmark et al., 2007). We analyzed the effect of SkQ1 and C12TPP on [Ca^2+^]_c_ changes and found that both SkQ1 and C12TPP statistically significantly suppressed the [Ca^2+^]_c_ increase induced by the combination of fMLP with CB, but not A23187 or zymosan (Figures 2A–G). It is important to note that the amplitude of increase in [Ca^2+^]_c_ induced by fMLP was approx. 3 times higher than that of A23187 and zymosan (Figure 2G). At the same time, the increase in [Ca^2+^]_c_ induced by A23187 lasted much longer, which probably explains the stronger stimulation of leukotriene synthesis compared to fMLP or zymosan (Figure 1). The difference between the effects of fMLP and A23187 likely reflects fMLP-induced opening of the store-operated Ca^2+^ channels (Itagaki et al., 2002; Tintinger and Anderson 2004), while A23187 additionally stimulates Ca^2+^ entry into the cytosol by increasing plasma membrane permeability (Mahomed and Anderson 2000). If so, then SkQ1 and C12TPP may act to deplete intracellular Ca^2+^ stores which prevents the store-operated channels from opening. In support of this, we have shown that SkQ1 and C12TPP by themselves cause a marked increase in [Ca^2+^]_c_ (Figure 2H).

C12TPP inhibits the fMLP-induced [Ca^2+^]_c_ increase to the same extent as SkQ1 but does not affect leukotriene synthesis. These data suggest that the effect of SkQ1 on fMLP-induced leukotriene synthesis is not entirely mediated by changes in [Ca^2+^]_c_. Any role of [Ca^2+^]_c_ alterations in SkQ1-dependent inhibition of leukotriene synthesis induced by A23187 or OZ can be ruled out.

### Activation of mitogen-activated protein kinases is upstream targets for SkQ1-dependent inhibition of leukotriene synthesis in neutrophils

**FIGURE 3:**
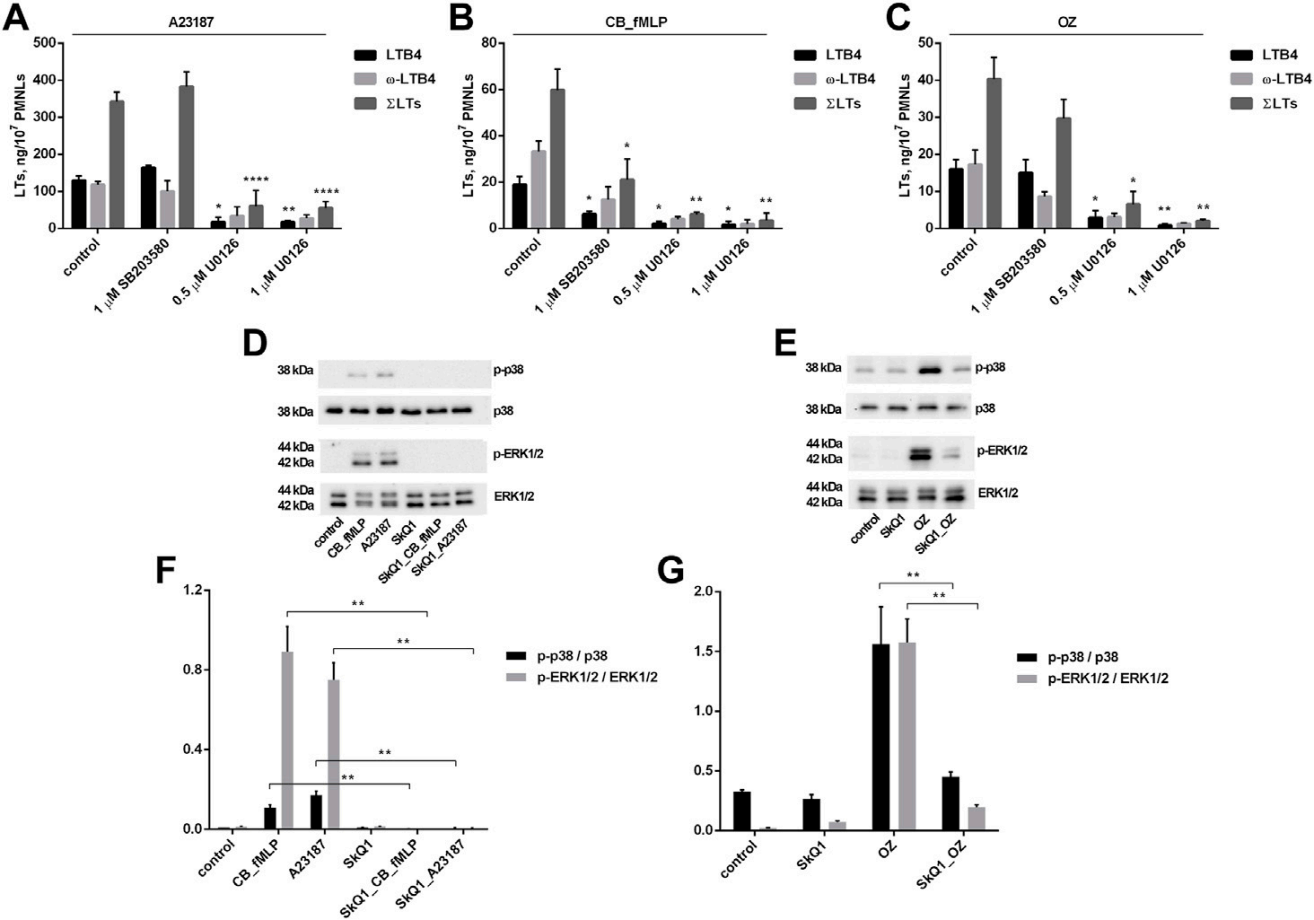
Effect of p38 inhibitor SB203580 and ERK1/2 inhibitor U0126 on leukotriene synthesis in human neutrophils **(A–C)** and effect of SkQ1 on p38 and ERK1/2 phosphorylation **(D–G)**. Before treatment, PMNLs [(1–1.2) × 10^7^/6 ml at **(A**–**C)**; 3 ml × 10^5^/0.3 ml at **(D**–**G)**] were pre-incubated for 10 min at 37°C at CO_2_ incubator. Then cells were treated 30 min with reagents indicated on *X*-axis **(A**–**C)** or with 100 nM SkQ1 (d,e,f,g, where indicated). After that, 1 µM A23187 (for 10 min) or, sequentially, 5 µM cytochalasin B (for 10 min) and 0.1 µM fMLP (for 10 min) or 0.04 mg/ml OZ (for 10 min), were added, as indicated. **(A–C)** The 5-LOX products were analyzed using HPLC, and data for LTB4, ω-OH-LTB4 and the sum of leukotrienes (ΣLTs = LTB4 + trans-LTB4 + ω-OH-LTB4) are presented. Values present mean ± SEM of three independent experiments performed in duplicate. *p < 0.05, **p < 0.01, ****p < 0.0001 for pairs of data (marked column compared with corresponding control value) by two-way ANOVA followed by Tukey’s multiple comparison test. **(D**–**G)** PMNLs lysates were separated onto 12% SDS polyacrylamide gels, then blotted to PVDF membranes and probed with antibodies against p-38, phospho-p38, ERK1/2, phospho-ERK1/2 as described in Methods. Representative Western blots are shown **(D,E)**. The ratios of phospho-p38 to p38 and phospho-ERK1/2 to ERK1/2 **(F,G)** are plotted as means ± SEM of three independent experiments, **p < 0.01 for pairs of data indicated by brackets.

To study the role of these kinases in the stimulation of leukotriene synthesis by the stimuli used above, we used the p38 inhibitor SB203580 and U0126, which inhibits the MAPK MEK kinase that activates ERK1/2. As shown in Figures 3A–C, SB203580 significantly inhibited leukotriene synthesis induced by fMLP in combination with CB but had no effect on synthesis induced by A23187 or zymosan. In contrast, U0126 almost completely suppressed leukotriene synthesis induced by any of the three stimuli.

Next, we analyzed whether p38 or ERK1/2 activation could be targets for modulation by SkQ1. SkQ1 was found to effectively prevent p38 and ERK1/2 phosphorylation (only phosphorylated forms of these kinases are active) induced by A23187 and CB/fMLP, and OZ (Figures 3D–G). These data suggest that activation of both kinases can be an upstream target for mtROS upon activation of leukotriene synthesis.

### Possible mechanisms of mtROS production responsible for synthesis of leukotrienes

**FIGURE 4:**
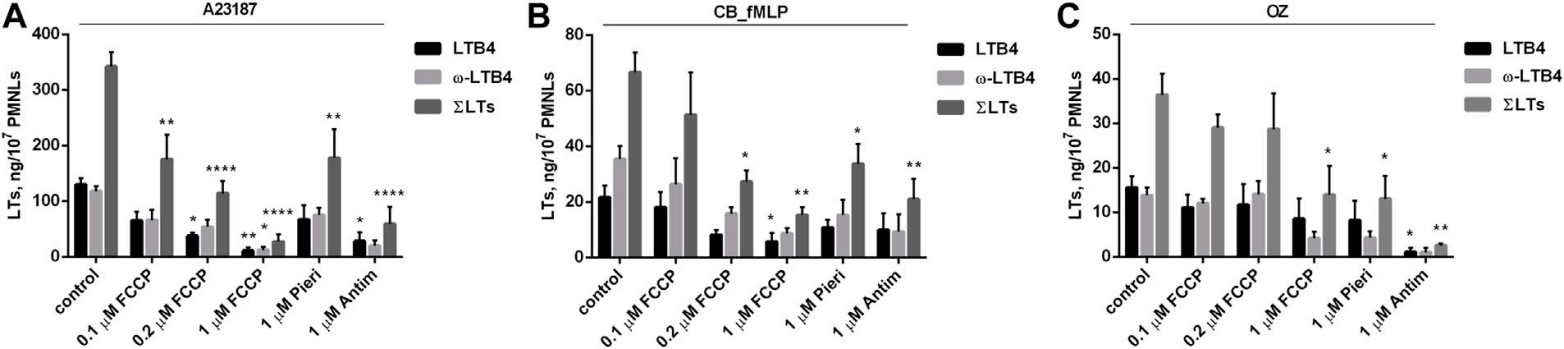
Effect of FCCP, Piericidin (Pieri) and Antimycin A (Antim) on leukotriene synthesis in human neutrophils. Before treatment, PMNLs (1–1.2) × 10^7^/6 ml were pre-incubated for 10 min at 37°C at CO_2_ incubator. Then cells were treated 30 min with reagents indicated on *X*-axis. After that, A23187 (1 µM) **(A)**, or cytochalasin B (5 µM) **(B)** or OZ (0.04 mg/ml) **(C)** were added for 10 min, then fMLP (0.1 µM) for 10 min **(B)**, as indicated. Values present mean ± SEM of three or more independent experiments performed in duplicate. The 5-LOX products were analyzed using HPLC, and data for LTB4, ω-OH-LTB4 and the sum of leukotrienes (ΣLTs = LTB4 + trans-LTB4 + ω-OH-LTB4) are presented. *p < 0.05, **p < 0.01, ****p < 0.0001 for pairs of data (marked column compared with corresponding control value) by two-way ANOVA followed by Tukey’s multiple comparison test.

The main sites for the production of ROS in mitochondria are complex I and complex III of the electron transport chain. In complex I, ROS can be formed either during the oxidation of NADH or during the energy-dependent reduction of NAD+ (reverse electron transfer, RET) (Vinogradov and Grivennikova 2016; Yin and O'Neill 2021). As shown in Figure 4, the complex I inhibitor piericidin only partially inhibits leukotriene synthesis induced by all tested stimuli. The effectiveness of piericidin (1 μM) as a respiration inhibitor was confirmed in experiments with highly respiring HeLa cells (data not shown). Thus, RET-dependent ROS production may be only partially responsible for the activation of leukotriene synthesis in neutrophils.

It can be assumed that the production of mtROS upon stimulation of neutrophils depends on the accumulation of Ca^2+^ in mitochondria which is driven by transmembrane electrical potential (ΔΨ) and mediated by the mitochondrial calcium uniporter (De Stefani et al., 2011). Multiple mechanisms of Ca^2+^-induced mtROS have not been fully elucidated (Adam-Vizi and Starkov 2010). We analyzed the effects of the uncoupler of oxidative phosphorylation carbonyl cyanide 4-(trifluoromethoxy)phenylhydrazone (FCCP) and the inhibitor of respiration antimycin A, which dissipate ΔΨ, and observed strong inhibition of leukotriene synthesis induced by all tested stimuli (Figure 4). These data suggest that ΔΨ-dependent accumulation of Ca^2+^ in mitochondria is important for stimulation of leukotriene synthesis.

In extreme cases, mitochondrial Ca^2+^ overload can open a large non-selective pore in the mitochondrial inner membrane (mitochondrial permeability transition pore, mPTP) and increase ROS production (Antonucci et al., 2021). Opening of the mPTP in neutrophils stimulated by fMLP or A23187 was demonstrated recently (Vorobjeva et al., 2020). As shown in Figures 1D–F, no effect of the mPTP inhibitor cyclosporine A was observed, indicating that the mPTP opening does not mediate mtROS production responsible for stimulation of leukotriene synthesis in neutrophils.

To further analyze the effect of FCCP, we measured the increase in cytoplasmic Ca^2+^ induced by various stimuli after pretreatment of neutrophils with FCCP. As shown in Figures 2A–G, FCCP slightly reduced the increase in cytoplasmic Ca^2+^ induced by A23187 and OZ, but strongly prevented the effect of CB_fMLP combination. Possible mechanisms for these effects of FCCP and their role in the inhibition of leukotriene synthesis need further study.

## Discussion

Neutrophils are traditionally considered as cells with a few mitochondria, the energetics of which strongly depends on glycolysis (Curi et al., 2020). For long time it was generally accepted that mitochondria in neutrophils are involved only in apoptosis among cellular programs. The first indications of important role of mtROS in neutrophils functions were presented by Daiber and coworkers (Kroller-Schon et al., 2014), who found that myxothiazol, the inhibitor of Complex III of respiratory chain stimulates NADPH oxidase in human neutrophils and this effect was blocked by mitochondria-targeted antioxidant MitoTEMPO. Later we have shown that activation of NADPH oxidase in neutrophils by fMLP (Vorobjeva et al., 2017) or by A23187 (Vorobjeva et al., 2020) are mediated by mtROS and blocked by mitochondria-targeted antioxidant SkQ1. The similar interplay between mtROS and NADPH oxidase was described also in endothelial cells stimulated with angiotensin II (Doughan et al., 2008), in smooth muscle cells subjected to hypoxia (Rathore et al., 2008), and in immortalized lymphoblast cell line activated with PMA (Dikalov et al., 2012). In our previous studies we demonstrated that mtROS are involved in degranulation of neutrophils (Vorobjeva et al., 2017) and in formation of neutrophil extracellular traps (NETs) (Vorobjeva et al., 2020) mostly due to activation of NADPH oxidase. Moreover, in neutrophils from the patients with chronic granulomatous disease (CGD) lacking active NADPH oxidase mtROS were found critical for A23187-dependent NETs formation demonstrating that some targets of mtROS aside NADPH oxidase are important for neutrophils functions.

In the present study we demonstrated that stimulation of leukotriene synthesis, which does not depend on activation of NADPH oxidase, is prevented by two mitochondria-targeted antioxidants (SkQ1 and MitoQ) and by the uncoupler of oxidative phosphorylation FCCP (Figure 1). The SkQ1 analogue lacking the antioxidant quinone moiety (C12TPP) was ineffective while non-targeted antioxidant Trolox inhibited the synthesis of leukotrienes at much higher concentrations than SkQ1. It was shown that SkQ1 at the concentrations that almost completely block leukotriene synthesis in intact neutrophils did not affect 5-LOX activity in cell homogenate (Supplementary Figure S2). These data suggest that ROS produced in mitochondria (mtROS) are required for the synthesis of leukotrienes in neutrophils. The effect of SkQ1 was mediated by inhibition of 5-LOX (catalyzing a rate-limiting reaction of LTB4 synthesis) rather than PLA2 (producing arachidonic acid for LTB4 synthesis) since application of exogenous arachidonic acid did not affect the inhibitory action of SkQ1 (Supplementary Figure S4).

An important mechanism of mtROS production in some cells, including macrophages, depends on reverse electron transfer (energy-dependent reduction of NAD+, RET) catalyzed by complex I of the electron transport chain (Vinogradov and Grivennikova 2016; Yin and O'Neill 2021). We have shown that the complex I inhibitor piericidin only partially inhibits leukotriene synthesis induced by all tested stimuli (Figure 4), indicating that RET may be only partially responsible for the activation of leukotriene synthesis in neutrophils.

Inhibition of leukotriene synthesis by SkQ1 was observed upon neutrophils activation with three different stimuli either mediated by the different receptors (fMLP and zymosan) or receptor independent (A23187). The common intermediate in signaling induced by these three stimuli is an increase in cytosolic Ca^2+^ concentration ([(Ca^2+^)_c_]. We suggested that mtROS production was stimulated at high [Ca^2+^]_c_ due to energy-dependent accumulation of Ca^2+^ in mitochondrial matrix. In agreement with this assumption dissipation of mitochondrial transmembrane potential with FCCP or antimycin A (an inhibitor of complex III of electron-transport chain) inhibited leukotriene synthesis (Figure 4).

An increase in [Ca^2+^]_c_ in the mitochondrial matrix can stimulate mtROS production through various mechanisms, including stimulation of NADH production by matrix dehydrogenases, stimulation of nitric oxide synthase, etc. (Adam-Vizi and Starkov 2010). The binding of Ca^2+^ to cardiolipin can strongly modulate the functions of the electron transport chain components and cause dissociation of cytochrome c from the membrane, slowing down the transfer of electrons from complex III to complex IV and, therefore, enhancing the formation of ROS (Brookes et al., 2004). Another mechanism for increased mtROS production depends on Ca^2+^-dependent opening of the mitochondrial permeability transition pore (mPTP) (Antonucci et al., 2021). It has been suggested that the opening of mPTP mediates myxothiazol-dependent activation of NADPH oxidase in neutrophils (Kroller-Schon et al., 2014). We recently showed that mPTP-dependent mtROS production mediates the activation of NADPH oxidase by fMLP and A23187 as well as A23187-induced NETs formation (Vorobjeva et al., 2020). Here, we demonstrated that the mPTP inhibitor cyclosporine A did not affect leukotriene synthesis (Figure 1). Thus, the production of mtROS, which is responsible for the activation of leukotriene synthesis, does not depend on the opening of mPTP, in contrast to the stimulation of NADPH oxidase and the formation of NETs. Probably, this difference reflects the different sensitivity of the neutrophil responses to mtROS. In support of this assumption, leukotriene synthesis was completely inhibited at 100 nM SkQ1 (Figure 1), while complete inhibition of NADPH oxidase was reached only at 600 nM SkQ1, and NETs formation was not fully inhibited even at this concentration (Vorobjeva et al., 2020).

In search of downstream targets of mtROS involved in activation of leukotriene synthesis, we analyzed the role of MAP kinases Erk1/2 and p38. Using selective inhibitors, we demonstrated that ERK1/2 activity is critical for activation of leukotriene synthesis induced by any of the three stimuli, while activity of p38 is required only for activation with fMLP (Figure 3). SkQ1 prevents p38 and ERK1/2 activation induced by A23187, fMLP, or OZ (Figures 3D–G) suggesting that activation of both kinases is dependent on mtROS production. Activation of MAP kinases by upstream kinases depends on ATP and the effect of SkQ1 could be potentially mediated by ATP depletion. However, ATP supply in neutrophils fully depends on glycolysis (Curi et al., 2020), so the depletion of ATP by SkQ1 seems unlikely.

Both ERK1/2 and p38 are activated by upstream kinases at a conserved threonine-glutamate (or proline)-tyrosine motifs, and complete activation requires both Tyr and Thr phosphorylation (Payne et al., 1991; Zarubin and Han 2005). Phosphotyrosine levels are regulated by a large family of protein-tyrosine phosphatases (PTPs) that have a conserved cysteine residue, which is essential for catalysis and very susceptible to oxidation (Salmeen and Barford 2005). Inhibition of PTPs by ROS (especially hydrogen peroxide) is a key event in many signaling pathways initiated by growth factors (Lambeth 2004), angiotensin II (Tabet et al., 2008), hypoxia/reoxygenation (Sandin et al., 2011), etc. In neutrophils PTPs are specifically activated *via* inhibitory receptors which terminate their responses (Futosi et al., 2013). Receptor PTPs are inactivated by ROS, which are transiently and locally generated within cells by NADPH oxidases (Lambeth 2004), while the sources of ROS that inactivates non-receptor PTPs may be different. MtROS can inactivate PTPs either *via* activation of NADPH oxidases or directly. An example of direct inhibition of PTPs (in particular, PTP with dual-specificity DUSP6) by mtROS and subsequent ERK1/2 stimulation was presented in cervical carcinoma cells (Shagieva et al., 2017). Another PTP, that is, directly inhibited by mtROS is PTEN (phosphatase and tensin homolog deleted on chromosome 10) (Kim et al., 2018). Interestingly, PTEN is one of the main targets of ROS, during activation of inflammatory NF-kB signaling leading in particular to expression of cyclooxygenase and production of prostanoids (St-Germain et al., 2004). The above data indicate that mtROS directly inhibits PTPs that counteracts activation of ERK1/2 and p38, thereby causing activation of leukotriene synthesis in neutrophils.

Phosphorylation of 5-LOX by MAPKs together with Ca^2+^ stimulates translocation of the enzyme from the cytoplasm to the nuclear membrane, where it colocalizes with cPLA2 and 5-LOX-activating protein (FLAP) (Radmark et al., 2007). Translocation of 5-LOX to the nuclear membrane makes an important contribution to its activation so it may be one of the targets for SkQ1-dependent inhibition of leukotriene synthesis. Our data do not allow us to exclude other targets. For example, the production of arachidonic acid hydroperoxides, which are involved in the synthesis of leukotrienes, can be inhibited by SkQ1.

Dysregulation of leukotriene synthesis has long been associated with various pathologies associated with excessive inflammation, including asthma (Gelfand 2017), cardiovascular disease (Haeggstrom 2018), ischemic and toxic acute kidney injury (Noiri et al., 2000; Deng et al., 2017), neurological (Saiwai et al., 2010), neurodegenerative (Giannopoulos et al., 2015), as well as ophthalmic diseases (Hirakata et al., 2020; Sanchez-Tabernero et al., 2021), including uveitis (Liao et al., 2006) and dry eye syndrome (Chistyakov et al., 2020). In recent years, many 5-LOX inhibitors have been considered, both synthesized and found in nature, but their clinical usefulness has not been tested. Zileuton (trade name Zyflo) is the only active 5-LOX inhibitor marketed worldwide for the treatment of asthma. Mitochondria-targeted antioxidants have been proposed as a promising therapy for almost the same range of pathologies listed above (Zielonka et al., 2017; Zinovkin and Zamyatnin 2019). SkQ1 has been successfully used in preclinical studies for the treatment of cardiovascular and renal diseases (Bakeeva et al., 2008; Plotnikov et al., 2019), and also demonstrated anti-inflammatory activity in acute bacterial infection (Plotnikov et al., 2013) and in the systemic inflammatory response syndrome (SIRS) model (Zakharova et al., 2017). The high efficiency of eye drops containing SkQ1 has been demonstrated not only in various models of eye diseases in animals (Neroev et al., 2008; Zernii et al., 2017), but also in a clinical study of dry eye syndrome (Brzheskiy et al., 2015). The coincidence of these two lists of pathologies suggests that inhibition of 5-LOX product synthesis by SkQ1 may contribute to the therapeutic efficacy of this compound. The use of mitochondria-targeted antioxidants may be an important component in the treatment of pathologies associated with dysregulation of leukotriene synthesis.

## Data Availability

The original contributions presented in the study are included in the article/Supplementary Material, further inquiries can be directed to the corresponding authors.

## Supplementary material

The Supplementary material for this article can be found online: https://www.frontiersin.org/articles/10.3389/fphar.2022.1023517/full#supplementary-material
