# Supplementary material for: Mitochondria-targeted antioxidant SkQ1 inhibits leukotriene synthesis in human neutrophils
Source: Front Pharmacol. 2022 Nov 24;13:1023517. doi: 10.3389/fphar.2022.1023517 (PMC9729262; doi:10.3389/fphar.2022.1023517)
Supplement: Supplementary file 1 [file Table1.DOCX]

Supplementary Material

Supplementary Figure 1. **Effect of SkQ1 and C12 on the synthesis 5-LOX products in human neutrophils induced by Ca2+ ionophore A23187**. Before treatment, PMNLs (1-1.2) x10^7^/6 ml were pre-incubated for 10 min at 37^o^C at CO_2_ incubator. Then cells were treated for 30 min with reagents indicated on X-axis. After that, A23187 (1 µM) was added for 10min. The 5-LOX products were analyzed using HPLC, and data for 5-HETE, LTB4, t-LTB4 and ω-OH-LTB4 are presented. Values present mean ± SEM of three independent experiments performed in duplicate. *p < 0.05, ****p < 0.0001 for pairs of data (marked column compared with corresponding control value) by two-way ANOVA followed by Tukey’s multiple comparison test.

Supplementary Figure 2. **Effect of SkQ1 on 5-LOX product synthesis in human neutrophils and in cell homogenates.** For intact PMNLs, cell incubations were performed as described for Supplementary Figure 1. For cell-free assay, cell homogenates were pre-incubated for 10 min at 37^o^C in CO_2_ incubator with reagents indicated on X-axis, then 20µM arachidonic acid were added to start 5-LOX, as described in the Methods, section 2.2. The 5-LOX products were analyzed using HPLC, and data presented as percent to corresponding controls, without SkQ1. Values are mean ± SEM of three independent experiments performed in duplicate. ***p < 0.001, ****p < 0.0001 for pairs of data (as compared with corresponding control value) by two-way ANOVA followed by Tukey’s multiple comparison test.

Supplementary Figure 3. **Effect of Diamide and SkQ1 on leukotriene synthesis in human neutrophils** induced by Ca^2+^ ionophore A23187. Before treatment, PMNLs (1-1.2) x10^7^/6 ml were pre-incubated for 10 min at 37^o^C at CO_2_ incubator. Then cells were treated 30 min with indicated concentrations of Diamide and SkQ1. After that, A23187 (1 µM) was added for 10min. The 5-LOX products were analyzed using HPLC, and data for the sum of leukotrienes (ΣLTs = LTB4 + trans-LTB4 + ω-OH-LTB4) presented as percent to the control without SkQ1 and without Diamide. Values present mean ± SEM of three independent experiments performed in duplicate. *p < 0.05, **p < 0.01, ****p < 0.0001 for pairs of data (marked column compared with control value w/o SkQ1 and w/o diamide), and ^###^p < 0.001 for pair of data (marked columns) by two-way ANOVA followed by Tukey’s multiple comparison test.

Supplementary Figure 4. **Effect of SkQ1 on leukotriene synthesis in human neutrophils** induced by Ca^2+^ ionophore A23187 and arachidonic acid (AA). Before treatment, PMNLs (1-1.2) x10^7^/6 ml were pre-incubated for 10 min at 37^o^C at CO_2_ incubator. Then cells were treated 30 min with reagents indicated on X-axis. After that, AA (20 µM) and A23187 (1 µM) were added for 10min. The 5-LOX products were analyzed using HPLC, and data for LTB4, ω-OH-LTB4 and the sum of leukotrienes (ΣLTs = LTB4 + trans-LTB4 + ω-OH-LTB4) are presented. Values present mean ± SEM of three independent experiments performed in duplicate. **p < 0.01, ***p < 0.001, ****p < 0.0001 for pairs of data (marked column compared with corresponding control value) by two-way ANOVA followed by Tukey’s multiple comparison test.
